# The Practice of Surgery: Embracing Minor Surgery, and the Application of Dressings, Etc., Etc.

**Published:** 1849-11

**Authors:** 


### ARTICLE VII.

*The Practice of Surgery: Embracing Minor Surgery and the Application of Dressings, etc., etc., etc.*—By John Hastings, M.D., U.S.N.; Fellow of the College of Physicians of Philadelphia; Member of the Philadelphia Medical Society; Lecturer on Surgical Anatomy and Operative Surgery, etc. With numerous Ilustrations. Philadelphia: Lindsay & Blakiston. 1850. pp. 479. 12 mo. (From the Publishers.)

There are many very excellent text books on Surgery from which the student may choose; and many devoted exclusively to Minor Surgery: nevertheless we think this volume a very useful one. In it the student will find the principles of surgery detailed in a simple and concise manner, and while there are of course many things omitted which are to be found in the larger treatises, he will find sufficient of really practical and valuable information on the subjects of surgical diseases, minor and operative surgery, to render him competent to manage cases that ordinarily come into the hands of the general practitioner. The section devoted to the consideration of Inflammation, embraces a good view of the modern doctrines and opinions on the subject. The portion devoted to operative surgery is written with great clearness and simplicity; every thing being made as plain as possible to the comprehension of the student. The wood cuts with which it is illustrated are correct and accurate delineations, and add no little to its value. The chapter on Venereal diseases embraces all the modern researches of Ricord, Acton and others; and although containing some opinions which might be subjects of discussion, exhibits about all that is known of this very important class of diseases. Military and Naval surgeons often have a good deal of leisure time on their hands, and are frequently placed in situations where they possess peculiar facilities for observation. Dr. Hastings seems to have turned these advantages to a good account; and the result is a really valuable and instructive volume. The typographical execution of the volume is of a very superior character, and is creditable to the celebrated publishing house from which it emanates.

